# Cell-free DNA in female infertility: from pathological mechanisms to clinical biomarkers

**DOI:** 10.1186/s13048-026-01999-x

**Published:** 2026-02-13

**Authors:** Ling Lin, Xinyue Zhang, Siyuan Liu, Hongshan Ge

**Affiliations:** 1 Reproduction Medicine Centre, The Affiliated Taizhou People’ Hospital of Nanjing Medical University, Taizhou, People’s Republic of China; 2 Graduate School, Nanjing Medical University, Nanjing, People’s Republic of China

**Keywords:** Female infertility, CfDNA, PCOS, EMs, POI

## Abstract

Female infertility, accounting for 40–50% of infertility cases globally, underscores the urgent need for non-invasive biomarkers to guide early intervention. Cell-free DNA (cfDNA)—released via apoptosis, necrosis, or active secretion—has emerged as a dynamic molecular reflector of tissue pathophysiology. This review synthesizes recent advances in cfDNA biology and their clinical applications in female infertility. Mechanistically, cfDNA drives inflammation, oxidative stress, and neutrophil extracellular traps (NETs), exacerbating dysfunction in polycystic ovary syndrome (PCOS), endometriosis (EMs), and premature ovarian insufficiency (POI). Clinically, cfDNA characteristics in follicular fluid or plasma serve as diagnostic-prognostic tools: reduced mitochondrial cfDNA (cf-mtDNA) in PCOS signifies oocyte mitochondrial damage; elevated long-fragment ratios in EMs/POI reflect chronic inflammation; and low cfDNA integrity in assisted reproductive technology (ART) predicts poor embryo quality. Despite challenges in standardization and ethics, future integration with multi-omics platforms is poised to translate cfDNA analysis into precision reproductive medicine.

## Introduction

Infertility is defined as the failure to achieve a clinical pregnancy after at least 12 months of regular, unprotected sexual intercourse [1]. Approximately 70 million reproductive-aged couples worldwide are affected by infertility [2]. Etiological classifications primarily include female factors (40–50%), male factors (30–40%), and unexplained infertility (UI), accounting for approximately 15% of cases. Among the pathogenic mechanisms of female infertility, tubal obstruction or dysfunction, ovulatory disorders, and endometriosis (EMs) constitute the primary pathological bases. Ovulatory disorders are most commonly represented by polycystic ovary syndrome (PCOS) and premature ovarian insufficiency (POI) [3].

Current infertility diagnosis faces dual challenges. On one hand, etiological heterogeneity necessitates a complex diagnostic process involving multi-system evaluation, encompassing reproductive endocrinology, anatomy, and immunometabolic factors [4]. On the other hand, early symptoms of female infertility (e.g., alterations in menstrual cycle regularity or flow) are non-specific and easily overlooked. Establishing an early screening system holds significant clinical value for implementing precise interventions and improving reproductive outcomes. An ideal screening protocol should meet core requirements such as minimal invasiveness, accessibility, cost-effectiveness, and high diagnostic accuracy. However, existing diagnostic technologies have significant limitations. For instance, while hysterosalpingography (HSG) is convenient, its diagnostic accuracy is operator-and patient-dependent. Although laparoscopy is the gold standard for pelvic assessment, its invasive nature precludes its use as a general screening tool. Diagnosing other etiologies often relies on the combined analysis of multimodal examinations (imaging + laboratory testing), which lack timeliness and cost-effectiveness [4]. Notably, UI diagnosed after conventional investigations lacks specific biomarkers to guide individualized treatment, and even UI patients undergoing assisted reproductive technology (ART) face challenges with low pregnancy rates [5]. Therefore, developing novel biomarker-based screening systems has become a critical research direction for enhancing early infertility diagnosis. In this context, Cell-free DNA (cfDNA) testing offers a minimally invasive approach that can provide dynamic molecular insights complementary to traditional anatomical and hormonal assessments.

CfDNA refers to double-stranded DNA fragments released into the circulation via programmed cell death (apoptosis), pathological necrosis, and active secretion mechanisms. Based on organellar origin, cfDNA can be classified into two main categories: cell-free nuclear DNA (cf-nDNA) and cell-free mitochondrial DNA (cf-mtDNA) [6]. In recent years, the value of cfDNA in disease diagnosis and treatment has been extensively elucidated. In metabolic diseases, plasma levels of 90 bp cfDNA fragments in patients with non-alcoholic fatty liver disease (NAFLD) are significantly higher than in healthy controls and positively correlate with disease activity and severity [7]. In oncology research, the quantity of cfDNA in epithelial ovarian cancer patients far exceeds that in benign and borderline controls, correlating with disease stage and subtype. Furthermore, analysis of p53 mutations in cfDNA aligns with p53 immunohistochemistry results in tumor tissues [8]. In reproductive disorders, cfDNA applications have primarily focused on non-invasive prenatal testing (NIPT) and preimplantation genetic testing (PGT). For example, NIPT efficiently screens for fetal chromosomal aneuploidies (e.g., trisomy 21) by analyzing fetal-derived cfDNA in maternal blood [9], while cfDNA in spent embryo culture medium (SECM) provides a novel pathway for non-invasive embryonic genetic analysis [10]. These studies highlight the broad potential of cfDNA as a novel biomarker in precision medicine.

However, a significant portion of existing research predominantly focuses on the instrumental value of cfDNA in prenatal and preimplantation contexts, with insufficient exploration of its role in the pathological mechanisms and individualized diagnosis and treatment of female infertility. Within the significantly heterogeneous field, current literature is often confined to single diseases or mechanisms, lacking a systematic integration of cfDNA across multiple infertility etiologies. Furthermore, its multidimensional value as a dynamic biomarker in disease classification, treatment response prediction, and prognostic assessment remains underexplored. This review aims to address these critical gaps by synthesizing the pathophysiological roles and diagnostic-prognostic potential of cfDNA specifically in female infertility.

## Biological characteristics of CfDNA

### Generation, clearance, and fundamental characteristics

The release of cfDNA is primarily driven by cell death (apoptosis or necrosis) or active secretion mechanisms [6]. Programmed cell death (apoptosis) is a major source of cfDNA. During apoptosis, nucleases cleave nuclear chromatin into monomeric or multimeric fragments of 180–200 bp, released into the circulation via exocytosis. The fragment length corresponds highly with the nucleosome structure (~ 147 bp) [6, 11]. Pathological necrosis represents another significant pathway, where disruption of cell membrane integrity leads to the passive release (i.e., unregulated leakage due to membrane rupture) of nuclear DNA and mitochondrial DNA (mtDNA), resulting in longer cfDNA fragments (> 1,000 bp) [6, 11]. Additionally, active secretion mechanisms from living cells are increasingly recognized; for instance, exosomes or microvesicles can encapsulate specific DNA fragments for active secretion into the extracellular space. This type of cfDNA may participate in immune regulation [6, 11, 12]. Notably, the dominant release mechanism differs across pathological states.

The molecular characteristics of cfDNA in biological fluids exhibit diversity and dynamism. Fragment length varies depending on the source mechanism with apoptosis generating nucleosome-sized fragments and necrosis or active secretion yielding longer fragments. The concentration cfDNA shows individual variation, with levels in healthy individuals typically at low nanogram per milliliter levels but can surge in pathological states [13]. CfDNA can originate from the nuclear genome (cf-nDNA) or mitochondrial genome (cf-mtDNA), the latter being particularly sensitive to oxidative stress [14, 15]. Furthermore, cfDNA exhibits tissue-specific methylation and fragmentation patterns that can inform about its cellular origin [7, 16, 17]. It is important to note that the relationship between cfDNA fragment length and disease type (e.g., cancer vs. chronic inflammation) is complex and context-dependent. While some studies associate long fragments with necrosis in cancer [11], and short fragments with apoptosis in inflammation [18], tumor-derived cfDNA can also show a high proportion of short fragments due to high turnover and enzymatic processing, whereas chronic inflammation may sometimes involve processes leading to longer fragments. This underscores the importance of integrating multiple cfDNA features for accurate interpretation.

CfDNA clearance maintains a dynamic equilibrium through several pathways: hepatic processing, renal excretion, degradation by plasma DNases (e.g., DNase I) with a short half-life (15–120 min), and uptake by immune cells and the vascular endothelium [19–27]. Impairment in these clearance mechanisms can lead to cfDNA accumulation, potentially exacerbating disease processes.

### Pathophysiological triggers and multi-omics analysis

**Fig. 1:**
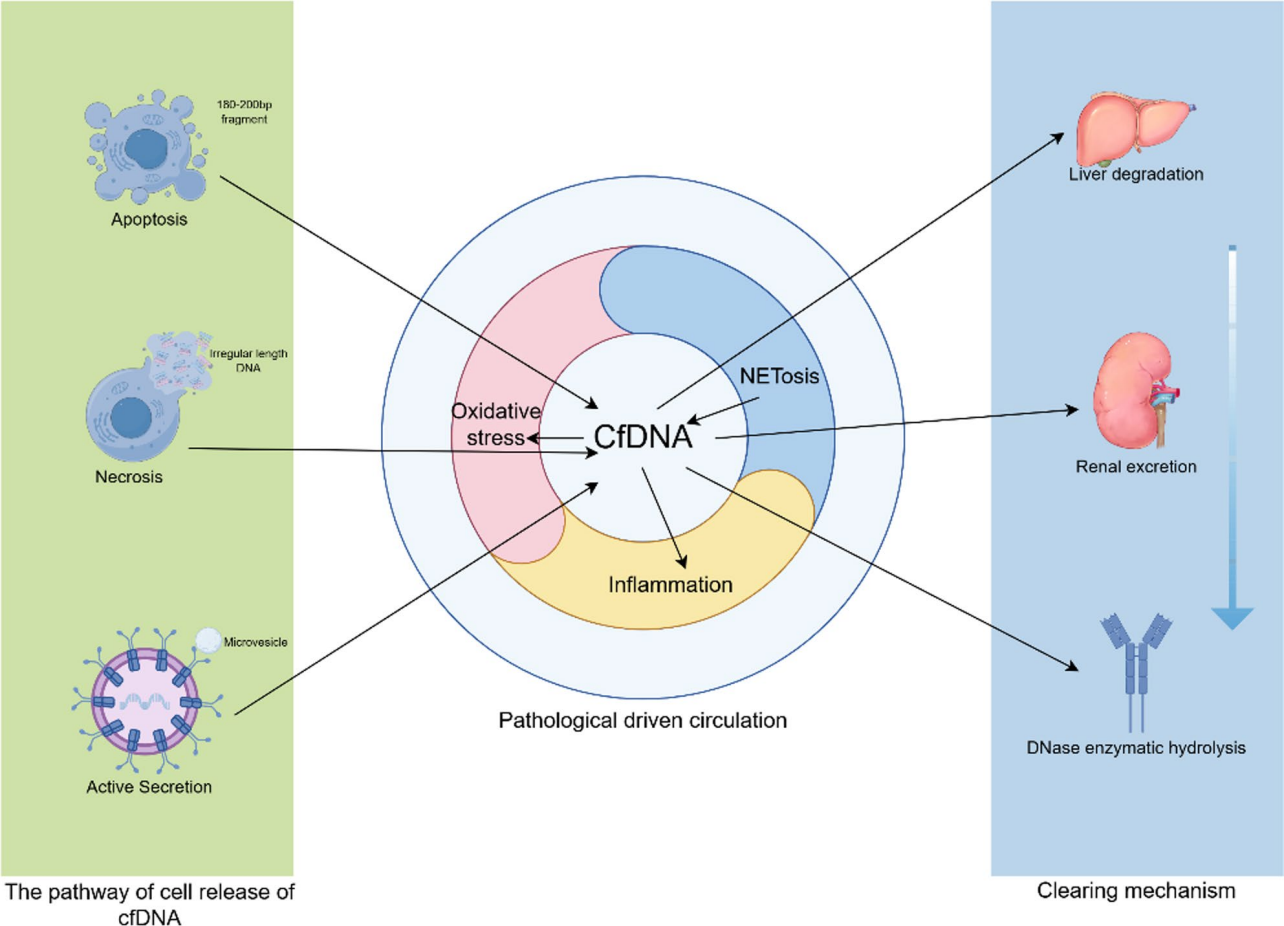
Sources, Clearance, and Pathological Triggers of cfDNA in Female Reprodcution

CfDNA release is amplified in pathophysiological states relevant to infertility. Under oxidative stress, reactive oxygen species (ROS) damage mitochondrial membranes, promoting mtDNA release into follicular fluid, as seen in PCOS [14, 28]. The inflammatory microenvironment in endometriosis enhances cellular apoptosis, increasing serum cfDNA [18]. Recent technological advances, such as Methylation Sequencing (MeD-seq) and nucleosome footprint profiling, have transformed cfDNA analysis from simple concentration measurement to multi-dimensional omics integration, significantly enhancing its diagnostic potential for infertility [29–33] (Fig. 1).

## Association mechanisms of CfDNA with female infertility

CfDNA, particularly mitochondrial-derived cf-mtDNA, can activate innate immune responses via pattern recognition receptors like Toll-like receptor 9 (TLR9), activating the NF-κB p65/MAPK p38 pathway and promoting pro-inflammatory cytokine release, thereby establishing a chronic inflammatory environment [34]. Both cf-nDNA and cf-mtDNA can contribute to these pathways, with cf-mtDNA being a potent inflammasome activator [28, 35]. Within the endometrium, such inflammation impairs stromal cell differentiation and receptivity [36]. In the ovary, it disrupts folliculogenesis and can accelerate atresia.

MtDNA released from damaged mitochondria forms a positive feedback loop with oxidative stress. Damaged mitochondria generate excess ROS, which further damages mitochondria, leading to more mtDNA release [14, 28]. ROS adversely affects oocyte quality, causing meiotic errors, and impairs endometrial integrity [36–38].

**Fig. 2:**
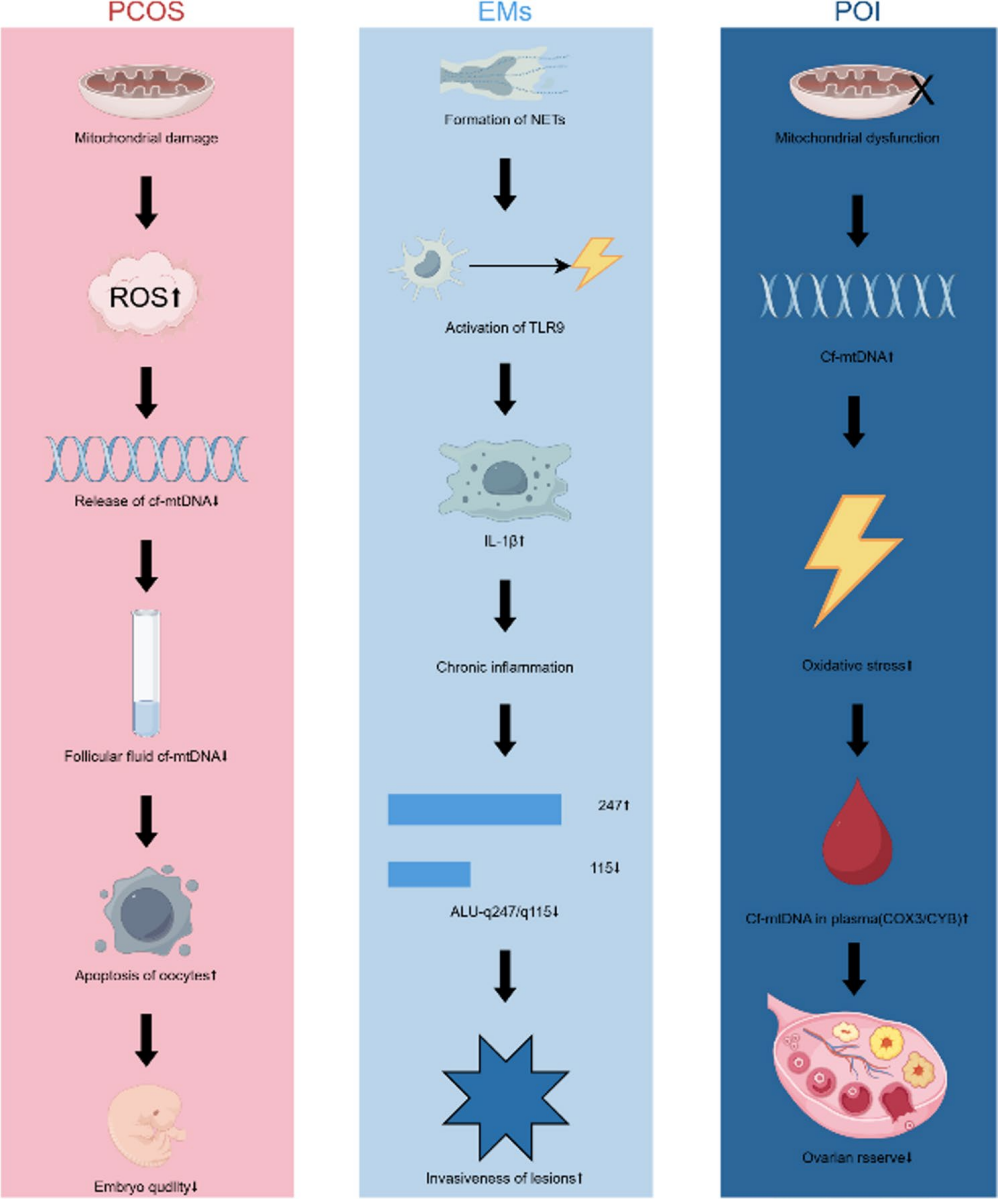
Disease-Specific cfDNA Pathogenic Loops in PCOS, EMs, and POI

Neutrophil Extracellular Traps (NETs), composed of cfDNA-histone complexes released by neutrophils, are another mechanism linking cfDNA to chronic inflammation [39]. Stimuli like hypoxia or autoantibodies induce NETosis. The cfDNA within NETs can perpetuate inflammation via TLR9. In EMs, elevated NETs formation contributes to the chronic inflammatory milieu and lesion progression [40, 41] (Fig. 2).

## Potential roles and challenges of CfDNA in evaluating oocyte and embryo quality

CfDNA demonstrates potential in assessing oocyte and embryo quality within ART. Embryonic-origin cfDNA in spent embryo culture medium (SECM) enables non-invasive screening for chromosomal aneuploidies, though challenges like maternal DNA contamination and embryonic mosaicism result in variable sensitivity compared to trophectoderm biopsy [10, 42, 43]. The analysis of cfDNA fragmentation patterns in SECM has also been correlated with embryo morphology and developmental potential [33].

Notably, maternal DNA contamination in SECM may cause false-positive results. Discarding potentially healthy embryos based on such results raises significant ethical concerns [42]. Moreover, cfDNA testing involves the acquisition and storage of embryonic genetic information, necessitating robust privacy protection frameworks. Technical bottlenecks also exist, including varying sensitivity/specificity across platforms and a lack of uniform quality control standards, which limit comparability [10, 43]. Addressing these challenges requires standardized protocols, algorithms to correct for contamination, and ethical guidelines.

## Clinical applications of CfDNA in infertility

### Diagnostic value

#### Diagnostic value in PCOS

The concentration characteristics of cfDNA in the follicular fluid of PCOS patients have shown initial potential as diagnostic markers, although research findings exhibit significant heterogeneity. Multiple studies report significantly higher total cfDNA concentrations in the follicular fluid of PCOS patients compared to controls [28, 44–46]. Furthermore, the relative copy number (RCN) of cf-mtDNA is significantly reduced in PCOS [28], suggesting mitochondrial dysfunction may be a key mechanism underlying poor oocyte quality. However, some studies found no significant difference in total cfDNA between PCOS and other infertility groups but observed an elevated long fragment ratio, indicating chronic inflammation as a common feature [47]. Age also influences cfDNA characteristics, providing a potential indicator for distinguishing physiological aging from PCOS-related dysfunction [48]. The impact of detection methods and disease heterogeneity on results is highlighted by studies showing no difference in specific methylated cfDNA markers [49]. In summary, cfDNA reflects the oxidative stress, mitochondrial damage, and inflammatory state of PCOS multidimensionally, though larger, standardized studies are needed.

#### Diagnostic value in EMs

Recent studies suggest that the concentration and fragmentation characteristics of cfDNA may provide novel molecular diagnostic clues for EMs. Serum cfDNA levels are elevated in EMs patients [50]. Follicular fluid cf-mtDNA concentration is significantly higher in patients with ovarian endometriotic cysts, implying mitochondrial damage may participate in EMs pathology [51]. Additionally, an elevated long fragment ratio in the follicular in EMs pathology [48]. Direct evidence of oxidative DNA damage in the follicular microenvironment of EMs patients comes from a study showing significantly higher concentrations of the biomarker 8-hydroxy-2’-deoxyguanosine (8OHdG) in their follicular fluid compared to infertile controls [45]. These findings collectively suggest that characteristics cfDNA changes may be linked to the inflammatory and oxidative microenvironment in EMs. Future research requires multicenter validation to confirm diagnostic sensitivity and specificity.

#### Diagnostic value in POI

Recent studies indicate that dynamic changes in cfDNA may offer a new approach for the molecular diagnosis of POI. Plasma levels of specific cf-mtDNA fragments are significantly higher in POI patients, suggesting abnormal mtDNA release may precede changes in traditional hormonal markers [37, 52–54]. Furthermore, a large nested case-control study demonstrated that a lower mtDNA copy number in peripheral blood leukocytes was strongly associated with subfertility (attempting pregnancy for 12–24 months), suggesting that peripheral blood mtDNA content could serve as a potential biomarker for diminished ovarian reserve or fecundity even before a formal POI diagnosis [53]. An elevated long fragment ratio in the follicular fluid of POI patients further reflects the impact of chronic tissue damage or an inflammatory microenvironment [47]. Although constrained by sample heterogeneity, cfDNA has the potential to become a novel tool for early POI screening (Table 1).

**Table 1:** Detailed comparison of CfDNA biomarker characteristics, mechanistic associations, and clinical significance in PCOS, EMs, and POI

Disease	cfDNA Feature & Sample Type	Core Mechanism & Pathophysiology	Clinical Significance & Key References
PCOS	↓ cf-mtDNA RCN (Follicular Fluid) ↑Total cfDNA conc. (Follicular Fluid) ↑Long fragment ratio (ALU-q247/q115)	Granulosa cell apoptosis, Mitochondrial dysfunction, Chronic low-grade inflammation	Assess oocyte mitochondrial health and oxidative stress level. Potential biomarker for oocyte quality and ovarian response. Refs: [29,45,48,49]
EMs	↑ cf-mtDNA conc. (Follicular Fluid) ↑ Total cfDNA conc. (Serum/Plasma) ↑ Long fragment ratio (ALU-q247/q115) ↑ NETs-associated cfDNA	NETs-mediated chronic inflammation, Mitochondrial damage in ectopic lesions, Apoptosis in inflammatory milieu	Aid in non-invasive screening and staging assessment. Reflects inflammatory burden and lesion activity. Monitor treatment response. Refs: [18,41,42,49,51,52]
POI	↑ cf-mtDNA levels (Plasma) ↑ Long fragment ratio (Follicular Fluid)	Mitochondrial dysfunction and biogenesis failure, Increased oxidative stress, Chronic tissue damage/apoptosis	Early screening of ovarian reserve decline before hormonal changes. Prognostic assessment and potential for subtype classification. Refs: [38,48]

### Age-related changes in CfDNA and their implications for reproductive aging

Recent studies have expanded our understanding of cfDNA dynamics to include physiological aging. Tessier et al. [55] reported a significant age-dependent increase in nuclear ccfDNA (ccfnDNA) levels remained stable. These findings suggest that aging itself is associated with enhanced cellular turnover and apoptosis. The age-related decline in cfDNA integrity mirrors patterns seen in chronic inflammatory states, underscoring the potential of cfDNA as a biomarker for both pathological and physiological reproductive aging, which may inform patient stratification.

### Therapeutic and prognostic value

CfDNA in follicular fluid shows significant prognostic potential in ART. Parameters like cfDNA integrity and concentration are closely related to embryonic developmental potential. High cfDNA integrity correlates with better embryo grade, while elevated concentrations are often associated with poor oocyte maturity and embryo quality [56–58].Furthermore, follicular fluid cf-mtDNA content negatively correlates with blastocyst formation rate [59].Studies specifically examining plasma cfDNA during in vitro fertilization (IVF) cycles have reported that elevated cfDNA levels on the day of embryo transfer or pregnancy test are associated with a lower probability of conception [52]. Interesting data also comes from studies on poor responders, comparing cfDNA in follicular (ff cfDNA) between follicular phase and luteal phase oocyte retrievals (LuPOR). One study found higher levels of apoptosis-associated cfDNA fragments in ff from LuPOR, and further observed that cfDNA integrity in ff was correlated with embryo development potential in both phases [54]. A small pilot study also suggested that stress reduction techniques might lower elevated plasma cfDNA levels in IVF patients, hinting at the dynamic nature of this biomarker and potential non-pharmacological intervention avenues [46].

These findings suggest cfDNA can serve as an auxiliary indicator of ovarian response and oocyte competence. While follicular fluid provides direct insight into the ovarian microenvironment, its collection is invasive. The potential for serum cfDNA to offer similar predictive value for ovarian responsiveness is an important area of future research. Current predictive value for pregnancy outcomes remains complex and heterogeneous, likely due to differences in cfDNA subtypes, sampling timing, and patient cohorts. Future research needs systematic exploration across different sampling timepoints and integration with multi-omics technologies to build precise predictive models.

## Summary and future perspectives

Infertility poses a significant global health burden. CfDNA, as a novel molecular biomarker, holds promise for reshaping its diagnosis and management. This review has synthesized how cfDNA is not merely a bystander but an active participant in the pathophysiology of key infertility disorders like PCOS, EMs and POI, through mechanisms including inflammation, oxidative stress, and NETosis. Clinically, cfDNA characteristics in biofluids like follicular fluid and plasma offer emerging diagnostic and prognostic tools, providing insights into ovarian reserve, oocyte quality, and embryonic potential that complement traditional methods.

Despite this promise, significant challenges remain. As highlighted in previous sections, current research is often constrained by small sample sizes, methodological heterogeneity, and a lack of standardized protocols for cfDNA analysis in reproductive contexts. The ethical implications, particularly regarding embryo selection based on SECM cfDNA analysis and genetic privacy, require careful multidisciplinary deliberation [29, 42].

Future efforts should focus on several key areas to translate potential into clinical utility, beginning with the conduction of large-scale, multicenter prospective studies to validate disease specific cfDNA thresholds and signatures. Furthermore, it will be essential to establish standardized pre-analytical and analytical protocols for cfDNA handling from relevant biofluids. Actively exploring the integration of cfDNA fragmentomics, methylation, and other features with complementary omics data, such as metabolomics, will also be critical to enhance diagnostic specificity and predictive power. Finally, developing ethical and regulatory frameworks to guide responsible innovation remains a foundational priority. Addressing these challenges will be crucial for driving a paradigm shift toward precision reproductive medicine and ultimately improving care for infertility patients worldwide.

## Data Availability

No datasets were generated or analysed during the current study.
